# Primary Immunodeficiencies: A Decade of Progress and a Promising Future

**DOI:** 10.3389/fimmu.2020.625753

**Published:** 2021-02-18

**Authors:** Isabelle Meyts, Aziz Bousfiha, Carla Duff, Surjit Singh, Yu Lung Lau, Antonio Condino-Neto, Liliana Bezrodnik, Adli Ali, Mehdi Adeli, Jose Drabwell

**Affiliations:** ^1^ Department of Pediatrics, Department of Microbiology, Immunology and Transplantation, Laboratory for Inborn Errors of Immunity, University Hospitals Leuven, KU Leuven, Leuven, Belgium; ^2^ European Society for Immunodeficiencies (ESID), Amsterdam, Netherlands; ^3^ Laboratory for Clinical Immunology, Inflammation and Allergy, Faculty of Medicine and Pharmacy, King Hassan II University, Casablanca, Morocco; ^4^ Clinical Immunology Unit, Pediatric Infectious Disease Department, Children’s Hospital, Ibn Rochd University Hospital, Casablanca, Morocco; ^5^ Department of Pediatrics, Division of Allergy and Immunology, Adjunct Clinical Faculty, College of Nursing, University of South Florida, Tampa, FL, United States; ^6^ International Nursing Group for Immunodeficiencies (INGID), Department of Laboratory Medicine, Karolinska Institute, Stockholm, Sweden; ^7^ Allergy Immunology Unit, Department of Pediatrics, Advanced Pediatrics Centre, Post Graduate Institute of Medical Education and Research (PGIMER), Chandigarh, India; ^8^ Indian Council of Medical Research (ICMR) Centre for Advanced Research in Primary Immunodeficiency Diseases, Chandigarh, India; ^9^ Asia Pacific Society for Immunodeficiencies (APSID), Department of Pediatrics and Adolescent Medicine, Queen Mary Hospital, Hong Kong, Hong Kong; ^10^ Department of Pediatrics and Adolescent Medicine, Queen Mary Hospital, Hong Kong, Hong Kong; ^11^ Department of Immunology, University of São Paulo, São Paulo, Brazil; ^12^ Department of Immunology, Jeffrey Model Centre Sao Paulo, Sao Paulo, Brazil; ^13^ Department of Immunology, Brazilian Society of Pediatrics, São Paulo, Brazil; ^14^ Latin American Society of Immunodeficiency (LASID), Department of Immunology, Mexico City, Mexico; ^15^ Center for Clinical Immunology, Immunology Working Group of the Ricardo Gutiérrez Hospital, Buenos Aires, Argentina; ^16^ Jeffrey Modell Centre Argentina, Clinical Immunology Center, Children’s Hospital, Buenos Aires, Argentina; ^17^ Department of Paediatrics, Faculty of Medicine UKM, Universiti Kebangsaan Malaysia (UKM) Medical Center, Kuala Lumpur, Malaysia; ^18^ Department of Immunology, Sidra Medicine, Doha, Qatar; ^19^ Department of Pediatrics, Weill Cornell Medicine, Doha, Qatar; ^20^ International Patient Organisation for Primary Immunodeficiencies (IPOPI), Ixelles, Belgium

**Keywords:** primary immunodeficiencies, awareness, immune system, immune deficiency, genetics, COVID-19

## Introduction

Around the world, over 6 million people are affected by primary immunodeficiencies, among which 70 to 90% remain undiagnosed (1). More than 430 different primary immunodeficiencies or primary immunodeficiency diseases (PIDs) have been described, caused by inherited defects in one or more component of the immune system. This leaves people living with PIDs more prone than other to infections but also to severe autoinflammation, autoimmunity, allergy, and malignancy (2, 3).

The ever-growing understanding of PIDs is crucial for future research and treatments, so that patients can enjoy an improved quality of life. The past decade has seen major advances in the field. However, many challenges still persist, some of which have been amplified by the COVID-19 crisis and need to be addressed in a collaborative way to allow another decade of progress.

## Overview of PIDs

PIDs are classified as rare diseases and cause a vulnerability to germs such as bacteria, viruses, fungi, and protozoa; infections that can turn chronic and generate long lasting healthcare issues or be fatal if they are not timely and adequately managed. PIDs can also predispose to cancer and immune diseases, including allergy, autoimmunity, and inflammation (2). Worldwide, approximately 1 in 10,000 people are affected by PIDs (2–5), a number which is likely underestimated due to “missed” diagnoses. The prevalence of the diseases varies greatly from country to country and is higher than this average in several countries. In France for instance, it is estimated that the overall prevalence is 4.4 cases per 100,000 inhabitants (6). The wide range of presentations of PIDs entail that some forms go undiagnosed for years. Symptoms vary according to the type of disease, however there are common signs that can alert to a potential primary immunodeficiency: increased susceptibility to infection, persistent disease, and in some cases specific organ—skin, heart, skeleton—problems (7). Treating only the systems without researching the underlying cause can lead to a deterioration of the patient’s health. Knowledge and awareness raising, notably among healthcare professionals, is therefore crucial. Especially the ever-growing spectrum of manifestations demands that physicians in all medical disciplines are aware of PID as their patient with neuromyelitis, inflammatory bowel disease, severe food allergy or early onset auto-immune disease or B cell lymphoma may well suffer from PID.

## Achievements in the Past 10 Years and the Ever-Growing Understanding of PID

Advances are particularly notable in the area of genetics, which has seen in recent years both the clinical application of gene addition strategies for treatment of PIDs (8) and the development of DNA sequencing technologies for diagnosis. Next generation sequencing (NGS) is considered a major breakthrough, faster, and cheaper than the conventional gene sequencing method especially when it comes to sequencing more than a handful of genes, which is often the case given the genetic heterogeneity so common to PIDs. Research efforts on disease-causing genes have been very active and have allowed to uncover a tremendous phenotypic diversity in PIDs. Over 400 distinct defects are now included in the latest International Union of Immunological Societies (IUIS) classification of 2019 (2), showing the variety of PIDs and their growing recognition. For years PIDs were defined by an increased sensibility to infections; though, numerous observations show that defects of immune genes can lead to clinical phenotypes unrelated to susceptibility to infection (2). This has led to an extended collaboration across medical disciplines. With greater understanding of the disease come new opportunities of treatment and care, a better future for patients. The bottleneck or caveat to the use of NGS results is the need for meticulous validation of variants prior to ascribing pathogenicity. This is the case not only for novel variants in novel PID genes but also for novel variants in known PID genes. Especially the validation of novel variants in novel PID genes is cumbersome and perceived as unattractive in the short term to researcher (9, 10).

Increased awareness of the warning signs—the strongest warning being family medical history—among the healthcare professional community has helped make progress in the diagnosis of PIDs. Further, new diagnosis protocols including screening for PIDs in medical specialties other than immunology have been set up (11). Nevertheless, these warning signs are mainly focused on the most common group of PID, namely the humoral immune deficiencies. The recognition of the expanding phenotype of PID calls for an update of the warning signs to aid non-immunologists in diagnosing PID. Recently, the expansion of newborn screening for Severe Combined Immunodeficiency’s (SCID)—one of the most severe forms of PIDs—also changed the disease landscape, as it allows for early detection (hence early treatment) and reduces the burden of infection (12). These past ten years the implementation of newborn screening has multiplied, including with widespread coverage throughout the United States. Additionally, recent joint stakeholders advocacy efforts have led to the inclusion of PID diagnostic tests in the WHO Model List of Essential *In Vitro* Diagnostics (13).

Remarkable improvements in therapies for PIDs have also been achieved, including in terms of allogeneic hematopoietic stem cell transplantation techniques, for instance with the use of post-transplant cyclophosphamide in T replete haplo-identical transplantation (14–16) which bypasses the need for expensive T cell depletion techniques; gene therapy successes (8) and best treatment approaches with intravenous immunoglobulins administration (IVIG) and subcutaneous immunoglobulins administration (SCIG). The use of biological treatments is also helping to manage several types of PIDs, as a substitute to conventional drugs which may come with co-morbidities and side effects. These developments are vital to improve patients’ quality of life and their long-term care. Nevertheless, treatment of these complex disorders remains challenging with many patients posing the dilemma of needing immune suppression to control inflammation whereas overall, the immune system is deficient in its functions.

## Challenges to be Addressed in the Next Decade

It will be crucial in the coming years to ensure universal access to the many advances we have seen, and provide a sustainable mechanism to allow timely access to future developments. While being a revolutionary way of diagnosing PIDs, next generation sequencing is not available in many countries, especially in low-income countries. Therefore, there is a real challenge to make this diagnosis technique universally available and affordable. Other rapid screening tests being developed for antibodies deficiencies, which have the potential to facilitate access to point-of-care testing in remote areas of less resourced countries, should also be made easily accessible. Further, new-born screening for SCID represents a hope for the early diagnosis and treatment of PIDs, but it needs to be implemented more broadly in public and private healthcare settings as it provides a chance to detect and cure PIDs early on (12). Following the United States, several European countries are now starting pilot implementation projects or have introduced neonatal screening, and this trend needs to continue in the rest of Europe and equally so in other regions of the world.

Inequalities in access to treatment and care including reimbursement issues, availability, access to care structures etc.—still persist across the globe and within regions and countries. Further, quantitative analysis of needs in care in the different regions of the world—and notably in Asia Pacific, is needed to support advocacy efforts for greater public investment in PIDs care and research (17).

As still too little is known about PIDs, they are often perceived as “exotic” conditions. Improving awareness, understanding, and the continuous recognition of new forms will offer the potential to change the lives of many other patients in the future. Collaborative work needs to be pursued to maintain plasma-derived medicinal product supplies across the world, including at times when healthcare systems experience strains on blood and plasma supplies.

The discovery of new, cutting-edge treatments is a continuous necessity as we uncover new disease types and understand better disease patterns.

## Learnings From the Covid-19 Crisis and Implications for PIDs

The current COVID-19 pandemic has underlined the fragility of our healthcare systems and put in jeopardy the fulfillment of some essential healthcare needs for primary immunodeficiency patients: in particular, a sustainable plasma supply to allow for continuity of treatment for patients who rely on immunoglobulin therapies, the most common type of treatment for PIDs. Social distancing measures and confinement restrictions have had a negative impact on plasma donation. Many countries have reported significant drops in blood collection since the beginning of the crisis. However, and despite findings from a Chinese study alerting on the detection of SARS-CoV-2 RNA in blood donations (18) there is no risk for primary immunodeficiency patients regarding transmission *via* immunoglobulin therapies as removal steps and virus inactivation procedures are made during the manufacturing process of plasma-derived products (19).

As patients with PIDs can be particularly vulnerable to infections and, as the hypothesis of a genetic predisposition to severe COVID-19 is further studied (monogenic inborn errors of immunity), the crisis has generated great concerns among the community (20). A large international survey on the outcome of SARS-CoV2 was recently published. More data and an in-depth study will be necessary to further define the patients at-risk of severe COVID-19 (21). Yet, this unprecedented crisis requires that new measures and actions be taken to protect them, including that PIDs be prioritized as an indication for immunoglobulin replacement therapies (which are recognized to be essential medicines for PIDs by the WHO) in case of plasma and products shortages (22). Further, vaccines against SARSCov2 may not work for people living with PIDs as many of them cannot react to vaccine antigens and will not develop T or B cell immunity. It will be necessary to work out alternative ways of protecting patients, for instance by developing either monoclonal or polyclonal antibodies against SARSCov2 and provide these on a regular basis to patients with T and or B cell deficiencies or ensure that immunoglobulins will contain protective antibodies against the virus.

Another challenge which has been exacerbated by the COVID-19 crisis is the difficulty to ensure equal access to care and specialist doctors for patients with PIDs—similarly to other patients with rare and chronic diseases. Around the world, 80% of primary immunodeficiency patients do not have access to adequate care. Indeed, in some parts of the world, sometimes almost no patients are diagnosed with PIDs, exposing stark disparities in diagnosis and care (23). This situation and the COVID-19 crisis with stay-at-home measures have further impacted access to and continuity of care for patients. On the other hand, access to home therapy, which is for instance possible with SCIG infusions, may reduce the exposure of patients with PIDs during this vulnerable period. However, in some regions such as South-East Asia, access to home treatment is very limited. Long-term solutions will have to be developed drawing on the lessons from the COVID-19 crisis to provide care and a sustainable supply of blood/plasma and essential medicines for patients, during crises and beyond. International, national, and regional authorities must act to combine and ensure the maintenance of plasma supplies, which are essential to produce certain types of primary immunodeficiency therapies, and to treat many other diseases.

Raising awareness of these genetic, hereditary defects of the immune system has taken on an even greater significance during the COVID-19 crisis. Paradoxically, as the general population experienced life with the omni-present risk of contracting an infection, the crisis may have helped to improve understanding of diseases of the immune system, such as PIDs, and promoted efforts encouraging people to protect each other. Indeed, for the first time it seems that all medical disciplines are interested in investigating why a previously seemingly healthy individual succumbs or almost succumbs to a viral infection, whereas this has been the diagnostic question for physicians involved in the care of PIDs for many decades (24, 25).

Six months into the pandemic, two “twin” publications in *Science* provide compelling evidence that a net deficiency in type I interferon underlies severe COVID-19 pneumonia in at least 13.4% of patients in a large global cohort (26, 27). In the first paper, 3.4% of patients were found to have pathogenic variants in genes crucial to the production and signaling of type I IFN (26). In the other paper, an auto-immune phenocopy of these genetic defects is described with over 10% of patients with severe COVID-19 *versus* 0 patients with mild COVID-19 having IgG neutralizing anti-type I IFN auto-antibodies (27). This finding, importantly, was inspired by the observation of severe COVID-19 in three patients with APS-1 due to pathogenic variants in AIRE, in whom anti-type I IFN antibodies are pathognomonic. Yet another in-depth study of inborn errors of immunity has shed crucial insight into the factors that govern human anti-microbial defense, even in the context of the current pandemic.

Meanwhile, patient organizations have played a vital role in providing social and psychological support to patients, as well as providing guidance and assisting patients who could no longer visit the hospitals or access their treatments.

## What the Future Holds

Primary immunodeficiency is a pioneer area of medicine. Recent advances, greater understanding and knowledge of the disease hold the promise of a better future for patients and their families. Nonetheless, expectations for the coming decade are high to address existing challenges. First of all, it is essential to continue to raise awareness, as the disease is still unknown by the general public and healthcare professionals alike. Knowledge sharing and further epidemiological and medical training are needed to ensure earlier diagnosis and better care, taking into consideration the divergent Human Development Index (HDI) related resources and training needs between regions and countries. For the Asia Pacific region, these needs have been identified by APSID which provides tailored educational strategies (17). Similarly, ensuring access to care in low-income countries requires a strengthened response. Supporting low-income countries to develop or increase capacities to locally manufacture current treatment options may be a way to improve availability and affordability. Innovative therapies have emerged, thanks to state-of-the-art technology and precision medicine, and an increasing number of breakthroughs can be expected in the coming years. Collaboration will be key to ensure uptake and cross fertilization of knowledge in all these areas and expand neonatal screening for PIDs worldwide.

While the 10th anniversary of the World Primary Immunodeficiency Week campaign provided the opportunity to celebrate a decade of progress and successes in the field of primary immunodeficiency, we can be proud of the many advances made in recent years. We must continue to work together to further educate, implement patient-centered and forward-looking healthcare policies; and allocate sustainable budgets for research, diagnosis, and treatment of PIDs worldwide.

## Author Contributions

IM, AB, CD, SS, YL, AC-N, LB, AA, MA, and JD wrote the paper and reviewed it. All authors contributed to the article and approved the submitted version.

## Funding

IM is a FWO Senior Clinical Researcher and is supported by FWO G0B5120N G0E8420N and by C16/18/007 of KU Leuven and by the Jeffrey Modell Foundation.

## Conflict of Interest

The authors declare that the research was conducted in the absence of any commercial or financial relationships that could be construed as a potential conflict of interest.
